# Multi-Omics Integration to Reveal the Mechanism of Hepatotoxicity Induced by Dictamnine

**DOI:** 10.3389/fcell.2021.700120

**Published:** 2021-09-14

**Authors:** Can Tu, Ziying Xu, Lichun Tian, Zihui Yu, Tieshang Wang, Zhaojuan Guo, Jingxuan Zhang, Ting Wang

**Affiliations:** ^1^Beijing Research Institute of Chinese Medicine, Beijing University of Chinese Medicine, Beijing, China; ^2^Key Laboratory of Genomics and Precision Medicine, Beijing Institute of Genomics, Chinese Academy of Sciences (CAS), Beijing, China

**Keywords:** dictamnine, multi-omics analysis, mechanism, herb induced liver injury, Roussel Uclaf causality assessment method

## Abstract

Herb-induced liver injury (HILI) has become a great concern worldwide due to the widespread usage of herbal products. Among these products is Dictamni Cortex (DC), a well-known Traditional Chinese Medicine (TCM), widely used to treat chronic dermatosis. Dictamni Cortex has drawn increasing attention because of its hepatotoxicity caused by the hepatotoxic component, dictamnine. However, the potential hepatotoxicity mechanism of dictamnine remains unclear. Therefore, this study aimed to use the multi-omics approach (transcriptomic, metabolomic, and proteomic analyses) to identify genes, metabolites, and proteins expressions associated with dictamnine-induced hepatotoxicity. A study on mice revealed that a high dose of dictamnine significantly increases serum aspartate aminotransferase (AST) activity, total bilirubin (TBIL), and direct bilirubin (DBIL) levels, the relative liver weight and liver/brain weight ratio in female mice (*P* < 0.05 and *P* < 0.01), compared to the normal control group. Liver histologic analysis further revealed a high dose of dictamnine on female mice caused hepatocyte vesicular steatosis characterized by hepatocyte microvesicles around the liver lobules. The expressed genes, proteins, and metabolites exhibited strong associations with lipid metabolism disorder and oxidative stress. Dictamnine caused increased oxidative stress and early hepatic apoptosis via up-regulation of glutathione S transferase a1 (GSTA1) and Bax/Bcl-2 ratio and down-regulation of the antioxidative enzymes superoxide dismutase (SOD), catalase, and glutathione peroxidase 1 (GPx-1). Besides, the up-regulation of Acyl-CoA synthetase long-chain family member 4 (ACSL4) and down-regulation of acetyl-coa acetyltransferase 1 (ACAT1) and fatty acid binding protein 1 (FABP-1) proteins were linked to lipid metabolism disorder. In summary, dictamnine induces dose-dependent hepatotoxicity in mice, which impairs lipid metabolism and aggravates oxidative stress.

## Introduction

Traditional Chinese medicine (TCM) and herbal medicines have been used to treat various diseases and ailments for thousands of years, particularly in East Asia (Ye and He, 2010; Liu et al., 2020). In recent years, the popularity of herbal remedies has spread to other parts of the world. However, the rapid increase in global usage of herbal medicine has raised concerns regarding the safety of herbal products (Liu et al., 2016). The potential toxicity of herbal drugs, particularly herb-induced liver injury (HILI), is a serious medical issue (Liu et al., 2020). Herb-related products are ranked second among the most common causes of hepatotoxicity in the US-based on the recent guideline published by the American College of Gastroenterology (Navarro et al., 2014; Chalasani et al., 2021). According to LiverTox, a database maintained by the US National Library of Medicine, over 30 herbal medicines cause this hepatotoxicity (Teschke et al., 2014; Jin et al., 2016). In China, herbal hepatotoxicity and diagnosis of HILI are documented from preclinical studies to clinical observations (Wang et al., 2015; Zhu et al., 2016; Gao et al., 2019). However, most HILI studies exist solely as case reports, with the herbal drugs suspected or known to cause HILI rarely listed due to the difficulties in carrying out HILI studies.

In clinical practice, the prevention and diagnosis of HILI are extremely difficult owing to the lack of test criteria for an accurate diagnosis and the limited understanding of HILI mechanisms. The Roussel Uclaf causality assessment method (RUCAM) is commonly used to assess the association between liver injury and the medications implicated to cause the injury (Rochon et al., 2008; Danan and Teschke, 2018; Chen et al., 2021). Reliable HILI diagnostic methods were recently established based on the integrated evidence from the chain-based identification of Chinese herbal medicines (Wang et al., 2015, 2018). The Chinese Food and Drug Administration (CFDA) officially published the clinical evaluation guidelines for TCM-induced liver injury in 2018 (Xiao et al., 2019). Advances have also been made to enhance the understanding of HILI, including their prevention and treatment approaches. Despite these strides, the mechanisms by which DILI occurs remains poorly understood (Björnsson, 2010).

Dictamni Cortex (DC), a well-known TCM, has been widely used in East Asia and Western countries to treat chronic dermatosis (Lv et al., 2015; Chang et al., 2016). Like most herbal medicines, DC lacks a systemic safety evaluation, including reports on occasional adverse effects, especially hepatotoxicity, which has increased significantly in the recent years (Wang et al., 2014; Zhang et al., 2016; Shi et al., 2019). For example, in the UK, where DC was used to treat chronic skin diseases, it was linked to acute hepatitis as a side effect in the 1990s (Kane et al., 1995). In Korea, DC was ranked the second most used herbal product causing HILI, with 33 (91.7%) cases having a cumulative RUCAM score above 7. One of the cases had a RUCAM score of 10, suggesting that the causality grading between liver injury and DC was probable (Lee et al., 2015). In China, a typical clinical case of liver injury following DC administration had a RUCAM score of 6 according to the guidance for the clinical evaluation of TCM-induced liver injury. The frequent cases of DC-related liver injury in China motivated the search for herbal authentications, adulterations, toxin contaminations, and a preliminary clinical diagnosis of HILI (Huang et al., 2017). As a result, “*Zhi-Xue* capsule,” a DC-containing Chinese patent medicine, was warned of security risk by CFDA in 2008 and withdrawn from the Chinese market due to its severe hepatotoxicity (Tai et al., 2010). Overall, the safety evaluation, rational drug use, and DC preparations in China still face significant challenges.

Research on the pathogenesis mechanisms of HILI has increased in recent years (Seeff et al., 2015; Navarro et al., 2017). Host gene polymorphism, drug metabolism, mitochondrial function damage, and immune factor-related hypotheses are now considered important, acceptable and references in explaining the susceptibility to hepatotoxicity (Hoofnagle and Bjornsson, 2019). The preliminary findings in our study revealed that aqueous and ethanol extracts of DC have potential hepatotoxicity. The hepatotoxic component in DC has been confirmed as dictamnine by liquid chromatography with tandem mass spectrometry (LC-MS/MS) (Fan et al., 2018). However, the precise pathogenesis mechanism of dictamnine-induced hepatotoxicity remains largely unexplored. Therefore, it is of great clinical interest to explore how to potentiate the risk prevention and control of dictamnine-related hepatotoxicity.

The integration of transcriptomics, proteomics, and metabolomics in system biology is used in toxicity mechanism studies with large-scale datasets. A comprehensive analysis of metabolomics and transcriptomics has revealed new biomarkers and toxicity mechanistic insights of Di-(2-ethylhexyl) phthalate (DEHP) exposures (Lu et al., 2020). In addition, integrated omics analysis revealed a novel immunophenotypic classification of hepatocellular carcinomas (HCCs) that facilitates prognostic prediction (Zhang et al., 2019). Therefore, a comprehensive analysis of proteomics, metabolomics, and transcriptomics is a powerful tool in understanding the mechanisms underlying dictamnine-induced hepatotoxicity.

The present study aims to explore the mechanisms of dictamnine-induced hepatotoxicity. This study identified new potential biomarkers related to the pathogenesis mechanism and brand-new cellular pathways involved in dictamnine-induced hepatotoxicity, especially lipid metabolism and oxidative stress pathway.

## Materials and Methods

### Chemicals and Reagents

Methanol and acetonitrile (UPLC grade) were purchased from Merck (Darmstadt, Germany). Other organic solvents and reagents were all HPLC grade and their purity was above 99.5%. The Dictamnine was obtained from Chengdu Bio-Technology Co. Ltd. (Chengdu, China). Ketoconazole (KTZ) and dimethyl sulfoxide (DMSO) were purchased from Sigma-Aldrich (St. Louis, MO, United States). HepG2 cells were purchased from China Infrastructure of Cell Line Resources (Beijing, China). The ToxInsight DILI cartridge was acquired from Thermo Fisher Scientific (Waltham, MA, United States). Dulbecco’s modified Eagle’s medium (DMEM) and foetal bovine serum (FBS) were purchased from Gibco (Grand Island, NY, United States). Penicillin, streptomycin, cyclosporine A, and aspirin were obtained from Sigma Aldrich (Saint Louis, MO, United States). The cell counting kit (CCK-8) was purchased from Dojindo (Tokyo, Japan). Liquid paraffin and neutral formalin were purchased from Beijing Chemical Works (Beijing, China).

### Animals and Experimental Design

Adult male and female ICR mice (18–20 g) were obtained from the Beijing Vital River Laboratory Animal Technology Co. Ltd. (Beijing, China). The mice were housed in groups based on their weight in humidity (40–70%) and temperature (20–25°C) controlled rooms. The rooms had a 12-h light/dark cycle, and the food and water were provided *ad libitum* during the experimental period. The experimental procedures were approved by the experimental animal ethics subcommittee of Beijing University of Chinese Medicine (BUCM-4-2019091801-3067). The protocol used was designed as per the National Institute of Health Guide for the Care and Use of Laboratory Animals guidelines.

Hepatotoxic effect study: the ICR mice were weighed then randomly assigned into eight groups (two control groups and six treated groups), each consisting of 10 males and 10 females. The controls were treated orally with distilled water, while the treated groups’ mice were treated with dictamnine (Dic) at the dosage of 0.6, 3, and 15 mg/kg/day. Clinical observations, food consumption, body weights, biochemical parameters, organ weights, and the histopathology of mice were examined at the end of 4 weeks the treatment period.

Verification of dictamnine-induced hepatotoxicity responses: the ICR female mice were randomly assigned into four groups (Con, Dic, KTZ, KTZ/Dic). The experimental procedure followed the protocols previously described by Shi (Shi et al., 2019) and Li (Li et al., 2018) with minor modifications. The mice were pretreated with KTZ in KTZ and KZT/Dic groups (75 mg/kg, intraperitoneally, in 0.5% CMC-Na) 1.5 h before the oral administration of dictamnine (200 mg/kg) for seven consecutive days for the groups Dic and KTZ/Dic. Blood and liver tissues were collected 24 h following the last treatment. The mice were sacrificed, and the livers quickly removed. The left lateral liver lobes were fixed with 10% neutral formalin for 48–72 h, embedded in paraffin after fixation, continuously sectioned at a thickness of 5 mm, stained with hematoxylin and eosin (H&E) used for histopathological examinations.

### Cell Culture and Reagents

HepaRG cells were obtained from ATCC (Manassas, VA). HepaRG cells were maintained in MEM medium. All media contained 10% fetal bovine serum (GIBCO, United States), penicillin G (100 U/ml), and streptomycin (100 μg/ml) (Sigma-Aldrich, United States). All cells were cultured in a humidified atmosphere, containing 5% CO2. The cells were treated with Dictamnine at 20, 50, and 100 μM for varying durations.

### Cell Proliferation, Apoptosis Assay, and Cell Culture Medium Biochemistry Analysis

Cell proliferation assay was performed on the “IncuCyte” ZOOM Live-Cell Analysis System (Essen Bioscience). Cell proliferation was measured by analyzing the occupied area (% confluence) of cell images over time. Apoptosis assay was performed by fluorescence activated cell sorting (FACS) with Annexin V-FITC/PI staining. The cell culture medium was collected from the cell cultured with dictamnine in various concentrations and control after incubating for 48 h. The parameters of the biochemistry assays: alanine transaminase (ALT), aspartate transaminase (AST), the biochemical parameters were detected using the pyruvic acid method with a CX4 Pro automatic biochemical analyzer (Beckman, Brea, CA).

### FACS (Apoptosis and Cell Cycle)

Genomic DNA was extracted from Qiagen using QIAamp DNA mini kit, Cat#: 51306, while RNA was extracted from Life Tech using (TRIzol^TM^ reagent, Cat#: 15596-018) following the manufacturers’ instructions. Cell samples were heated at 95°C for 10 min on a heating block for denaturation then chilled on ice for 5 min. The chilled samples were dried through suctioning on a wet Hybrid Membrane prepared using the ECL kit (GE Amersham, Cat#: RPN2232). Thereafter, the membrane was heated at 80°C for 2 h, after which it was sealed with 5% non-fat milk in TBST (20 mM Tris-HCl, pH 7.4; 150 mM NaCl; 0.1% Tween-20) and maintained for 1 h at room temperature (RT). The membrane was then incubated with the primary antibody against 5hmC (dilution 1:10 000, Active motif) or 5mC (dilution 1:3 000, Active motif) for 1 h at RT. After incubation, the anti-rabbit or anti-mouse IgG-HRP antibody was added to the membrane and maintained for 30 min at RT.

### High-Content Screening Analysis (HCS)

For toxicity assessment, the cells were seeded in 96-well plates to a density of 4000 cells/well and maintained for 24 h in an incubator. The cells were then treated with dictamnine at a dosage of 20,100 μM and maintained in a DMSO (1% (v/v) supplemented culture medium. After 48 h incubation, the culture medium was aspirated and carefully replaced with 100 μl PBS cocktail containing 2.5 μM Cell-Rox Deep Red, 2 μg/ml BODIPY-green, 1 μg/ml Hoechst 33342 (Invitrogen), or a cocktail of 200 nM TMRE (Abcam), 20 μM Thiol Tracker violet, 1 μg/ml Hoechst 33342 (all from Invitrogen). The plates were then incubated for 30 min at 37°C, after which the cells were imaged using INCELL6 (GE Healthcare, Barcelona, Spain). The obtained images were analyzed using the INCELL workstation analysis module.

### Western Blotting

The total proteins in the liver tissues were prepared using ice-cold RIPA buffer and quantified using BCA Protein Quantification Kit (Vazyme, China) following the manufacturers’ instructions. Equal amounts of proteins were separated by SDS-PAGE, after which they were transferred into a polyvinylidene fluoride membrane prepared using ECL Kit (Xinsaimei, China). The membrane was then incubated with the primary antibody, followed by the addition of an anti-rabbit or anti-mouse IgG-HRP antibody. The antibodies used in the assay are listed in Supplementary Table 1.

### RNA Isolation and Real-Time RT-PCR

Liver tissues were disrupted in liquid nitrogen, followed by total RNA extraction using Trizol Reagent (Invitrogen, United States) according to the manufacturers’ instructions. After quantification, equivalent amounts of RNA were reverse transcribed using HiScript II Q Select RT SuperMix for qPCR (+ gDNA wiper) (Vazyme, China). Real-time RT-PCR was performed on the CFX96 Touch system detection system (Bio-Rad) using ChamQTMSYBRqPCR^®^ Master Mix (Vazyme, China) and primers listed in Supplementary Table 2. Gene expression analysis was performed using the comparative ΔΔCT method with GAPDH for normalization.

### Multi-Omics Analysis Method

The multi-omics (transcriptomic, metabolomic and proteomic) analyses were carried out to identify the expressions of genes, metabolites and proteins associated with the hepatotoxicity mechanism of dictamnine. Several assays are described in the Supplementary Material.

### Statistical Analysis

Statistical analysis was performed using SPSS (version 20.0) statistical analysis program. Raw data were subjected to a one-way analysis of variance (ANOVA), and the means were separated using Dunnett’s multiple comparisons test. Results were presented as mean ± SD, and *P* < 0.05 was considered significant.

## Results

### *In vitro* and *in vivo* Hepatotoxicity Effects of Dictamnine

In female mice treated with a high dose (15 mg/kg/day) of dictamnine, the serum AST activity was significantly increased compared to the control group (Table 1). In contrast, no significant differences in AST and ALT were observed between the dictamnine-treated male mice and the control. The high dose dictamnine treatment also caused a significant increase (*P* < 0.05 and *P* < 0.01) on the serum total bilirubin (TBIL), direct bilirubin (DBIL) and indirect bilirubin (IBIL) contents. In mice treated with 3 and 0.6 mg/kg/day of dictamnine, a significant increase in albumin to globulin ratio (A/G) and globulin (GLB) content compared to the control was observed. In addition, female mice treated with 15 mg/kg/day recorded a significantly high relative liver weight and liver/brain weight ratio when compared to the control group (*P* < 0.01) (Supplementary Table 3). In male mice, a significantly elevated (*P* < 0.05 and *P* < 0.01) relative liver weight ratio was documented both at 15 and 0.6 mg/kg/day dictamnine treated levels compared to the control.

**TABLE 1:** Effects of oral administration of dictamnine on the biochemical parameters in mice.

Index	Female(Dic,mg/kg)				Male(Dic,mg/kg)			
	Control	15	3	0.6	Control	15	3	0.6
A/G	1.30 ± 0.04	1.34 ± 0.08	1.45 ± 0.11**	1.43 ± 0.13*	1.13 ± 0.04	1.23 ± 0.06**	1.2 ± 0.06*	1.12 ± 0.1
ALB(g/L)	32.80 ± 11.32	32.8 ± 0.93	33.50 ± 91.3	33.69 ± 2.61	31.18 ± 1.86	31.52 ± 1.55	31.75 ± 1.5	31.79 ± 1.78
GLB(g/L)	25.17 ± 1.11	24.59 ± 1.85	23.34 ± 1.53*	23.65 ± 1.51*	27.58 ± 1.09	25.59 ± 1.68*	26.59 ± 1.72	28.52 ± 1.63
ALT(U/L)	31.86 ± 4.97	37.51 ± 1.77	33.5 ± 5.52	40.31 ± 3.02	37.00 ± 8.25	34 ± 5.42	48.00 ± 36.17	41.91 ± 0.93
AST(U/L)	116.51 ± 6.27	151.75 ± 45.29*	135.28 ± 25.59	135.54 ± 23.55	118.53 ± 22.56	121.73 ± 30.42	131.64 ± 33.22	125.46 ± 23.03
ALP(U/L)	128.21 ± 32.27	128.85 ± 30.88	152.66 ± 29.33	144.56 ± 32.11	140.66 ± 41.59	106.61 ± 11.52	120.17 ± 30.44	116.30 ± 22.05
TBIL(μM)	2.73 ± 0.67	3.66 ± 0.69*	3.01 ± 0.49	2.61 ± 0.86	4.43 ± 0.71	5.38 ± 2.50	5.24 ± 0.51*	5.13 ± 1.49
DBIL(μM)	0.56 ± 0.14	0.81 ± 0.18**	0.63 ± 0.13	0.59 ± 0.16	0.89 ± 0.06	1.13 ± 0.97	1.05 ± 0.16*	0.99 ± 0.27
IBIL(μM)	2.17 ± 0.56	2.85 ± 0.56*	2.38 ± 0.39	2.01 ± 0.77	3.54 ± 0.67	4.25 ± 1.55	4.19 ± 0.45	4.14 ± 1.34
TG (mM)	0.97 ± 0.45	0.99 ± 0.18	1.14 ± 0.10	1.11 ± 0.16	1.13 ± 0.29	1.29 ± 0.28	1.39 ± 0.38	1.61 ± 0.41
TP (mM)	57.98 ± 2.26	57.39 ± 2.42	56.93 ± 2.07	57.34 ± 3.16	58.75 ± 2.84	57.12 ± 2.97	58.34 ± 2.92	60.31 ± 2.03

Values are expressed as mean ± SD; n = 10; * P < 0.05, ** P < 0.01, compared with the control group.

**FIGURE 1:**
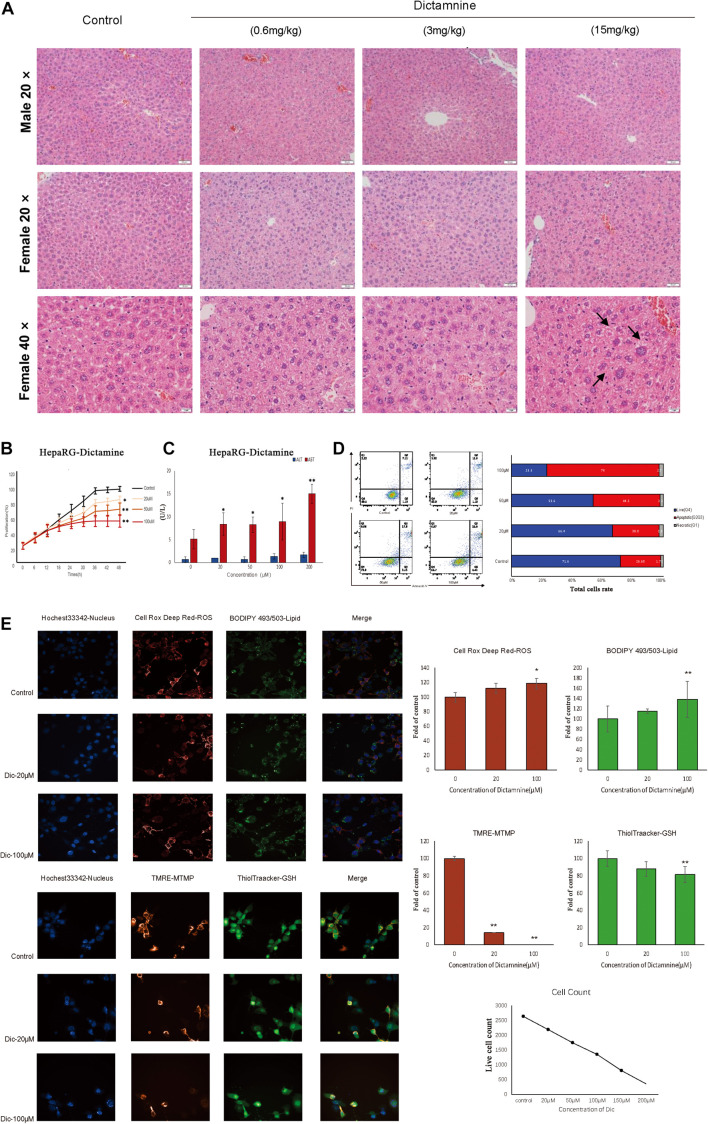
*In vivo* and *in vitro* dictamnine-induced hepatotoxicity. **(A)** Histological examination of livers of mice treated with dictamnine (H&E stained, 20, 40× magnification). **(B)** Apoptosis assay of HepaRG cells treated with multiple concentrations (20, 50, and 100 μM) of dictamnine. **(C)** Determination of ALT and AST biomarkers activities in dictamnine treated HepaRG cell lines. **(D)** High-content screening of HepaRG cells exposed to dictamnine (20 μM, 100 μM). ^∗^*P* < 0.05 and ^∗∗^*P* < 0.01, *vs.* Con). **(E)** Fluorescence of Hoechst 33342 (to stain nuclei; blue), Cell Deep Red (to reactive oxygen species production; deep red) and BODIPY493/503 (to stain neutral lipids; green); or fluorescence of Hoechst 33342 (to stain nuclei; blue), TMRE (to detect changes in mitochondrial membrane potential; red) and ThiolTracker (to stain glutathione; green).

Histological analysis of the liver revealed that liver injury was manifested as hepatocyte vesicular steatosis characterized by hepatocyte microvesicles around the liver lobules, hepatocellular microvesicles and cell enlargement in the high-dose dictamnine-treated female mice. No significant histologic lesions were observed in the control, medium and low dose dictamnine-treated groups (Figure 1A).

We did *in vitro* validation in cells. Hepatic cell lines HepaRG were treated with 20, 50, and 100 μM of dictamnine for 48 h. The proliferation of HepaRG cells was inhibited in a dose-dependent manner after a 48 h treatment with dictamnine compared to the control (Figure 1B). The AST activity was significantly increased in the dictamnine-treated HepaRG cells, compared to the control group (Figure 1C). The apoptosis assay further revealed that a high dose of dictamnine induced significant HepaRG-cell apoptosis (Figure 1D). In addition, the results showed that the number of nuclei decreased at a dose-dependent manner in dictamnine-treated cells, also demonstrated in the proliferation assay. Moreover, the reactive oxygen species (ROS) levels and lipids accumulation in cells increased with the increment of dictamnine concentration. Similarly, the mitochondrial membrane potential (MTMP) intensity and glutathione levels were proportionate to the dictamnine concentration (Figure 1E).

### Genome-Wide Transcriptome Analysis

**FIGURE 2:**
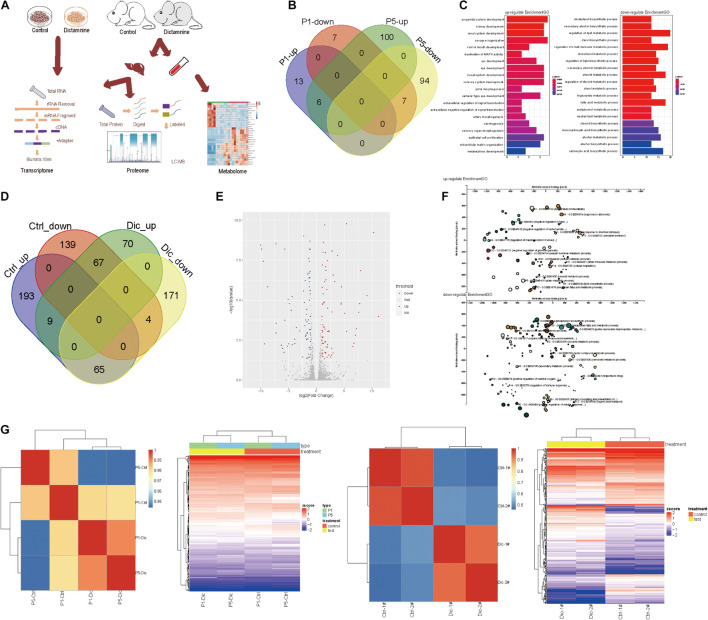
Transcriptome analysis of dictamnine-induced hepatotoxicity *in vitro and in vivo.* **(A)** Overview of methodology, animal samples collection (serum and liver tissue) and analyses. **(B)** Veen diagram display up-regulated and down-regulated genes in hepatic cells treated with dictamnine concentrated in PI and P5 cells. **(C)** The enriched GO terms of the RNA-seq data of HepaRG cells treated with dictamnine concentrated in PI and P5 cells. **(D)** Veen diagram display up-regulated and down-regulated genes in dictamnine treated female and male mice. **(E)** The volcano map of differentially expressed genes in dictamnine-treated female mice. **(F)** The enriched GO terms of RNA-seq data of dictamnine-treated female ICR mice. **(G)** Heatmap showing the expression level of differentially expressed in HepaRG cells (P1 vs. P5) and ICR mice (Ctrl vs. Dic), respectively.

To determine the effect of dictamnine on gene, protein and metabolite expression, genome-wide transcriptome, proteome and metabolome analyses were performed on HepaRG cells and ICR mice samples (serum and liver tissues). For the *In vitro* experiment, HepaRG cell samples were assigned into three groups: dictamnine treated-1-passage (P1) group, dictamnine treated-5-passage (P5) group, and untreated control. *In vivo*, the liver tissues and serum with and without dictamnine treatment were collected for genome-wide transcriptome, proteome, and metabolome profiling, respectively (Figure 2A).

*In vitro*, 33 differentially expressed genes were identified in P1 vs. control, of which 19 were up-regulated and 14 down-regulated in P5 vs. control, 207 genes were differentially expressed, with 106 up-regulated and 101 down-regulated. In total, six up-regulated and seven down-regulated genes were differentially expressed in both P1 *vs.* control and P5 *vs.* control. The differentially expressed genes in P1vs. control and P5 vs. control were pooled and subjected to a gene ontology (GO) and KEGG analysis. The differentially expressed genes were linked to lipid synthesis and lipid metabolic pathways (Figures 2B,C and Supplementary Figure 1A).

Interestingly, female mice were more sensitive to dictamnine than their male counterparts (Supplementary Figures 1B,C), as demonstrated by the gender-specific differences following dictamnine treatment (Figure 2C). A total of 70 up-regulated - and 171 down-regulated genes were identified in female vs. male mice following a dictamnine treatment (Figure 2D). Differential expression of genes was quantified using “*P* < 0.05, fold change > 1.0” as a cutoff level of significance (Figure 2E). Gene ontology and KEGG analysis revealed that the differentially expressed genes in female *vs.* male mice are associated with lipid synthesis, and drug metabolism pathways, etc. (Figure 2F and Supplementary Figures 1E,F).

Hierarchical clustering analysis of the differentially expressed genes *in vitro* and *in vivo* revealed a similarity in gene expression patterns of dictamnine-treated cells and mice (i.e., P1 and P5, and Dic1 and Dic2) but differed significantly from the Controls (Figure 2G).

### Metabolomic Profile Analysis of Dictamnine Related Hepatotoxicity

**FIGURE 3:**
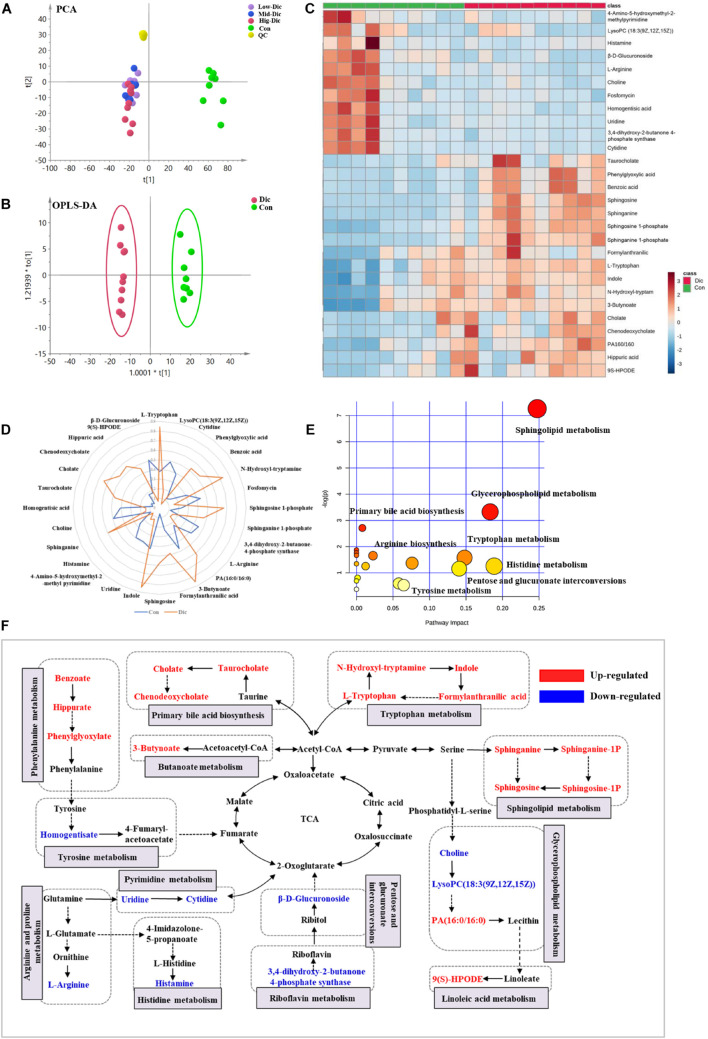
Metabolomic analysis of dictamnine-induced hepatotoxicity in mice. **(A)** PCA score plot for all groups female mice in ESI modes. **(B)** OPLS-DA score plot for experimental groups (Con *vs.* Dic). **(C)** Clustered heat map of the significantly differentially expressed metabolites between dictamnine-treated mice and untreated control. The colors from blue to red indicate the relative contents of metabolites. **(D)** A spider plot of 28 differentially expressed metabolites between Con *vs.* Dic groups. The blue line represents the control group, and the orange line represents Dic group. **(E)** Schematic diagram of the altered metabolic pathways. **(F)** Network map of metabolic pathways and metabolites in dictamnine treated and untreated control mice. Metabolites in red increased, and metabolites in blue decreased.

Unbiased metabolomics analysis was performed on serum samples collected from dictamnine-treated and untreated mice to identify the metabolites involved in dictamnine-induced hepatotoxicity. The unsupervised PCA and OPLS-DA score plots revealed distinct metabolite profiles between dictamnine-treated and untreated controls (Figures 3A,B) using multivariate and univariate statistical significance criteria (VIP > 1, Fold change > 1.5 or < 0.5 and *P* < 0.05). In total, 28 differentially expressed metabolites were identified, of which 17 were up-regulated (Supplementary Table 4). The pattern overview of metabolic alterations with the development of liver injury in a clinical setting is presented in a heatmap Figure 3C. In addition, the quantitative differences in the differentially expressed metabolites are presented in a radar chart (Figure 3D). Pathway enrichment analysis revealed that the differentially expressed metabolites were involved in the primary bile acid biosynthesis, sphingolipid metabolism, glycerophospholipid metabolism, etc., which highly correlated with the dictamnine -induced hepatotoxicity (Figure 3E). Finally, a network map was constructed based on the identified differential metabolites and enriched metabolic pathways. The interactions between the metabolic pathways and differentially expressed metabolites formed a complex network based on network analysis (Figure 3F).

A receiver operating characteristic (ROC) curve analysis was performed to further explore the diagnostic efficacy of differentially expressed metabolites. Based on the ROC curve analysis, differentially expressed metabolites, including chenodeoxycholate, taurocholate, and cholate, recorded excellent discrimination between the two groups (area under the curve (AUC) of ≥0.9), and may be used as potential biomarkers Supplementary Figure 2C. The ROC curves of the other differentially expressed metabolites ranked by the AUC are presented in Supplementary Figure 2A.

### Comprehensive Proteomics Characterization of Dictamnine-Related Hepatotoxicity

**FIGURE 4:**
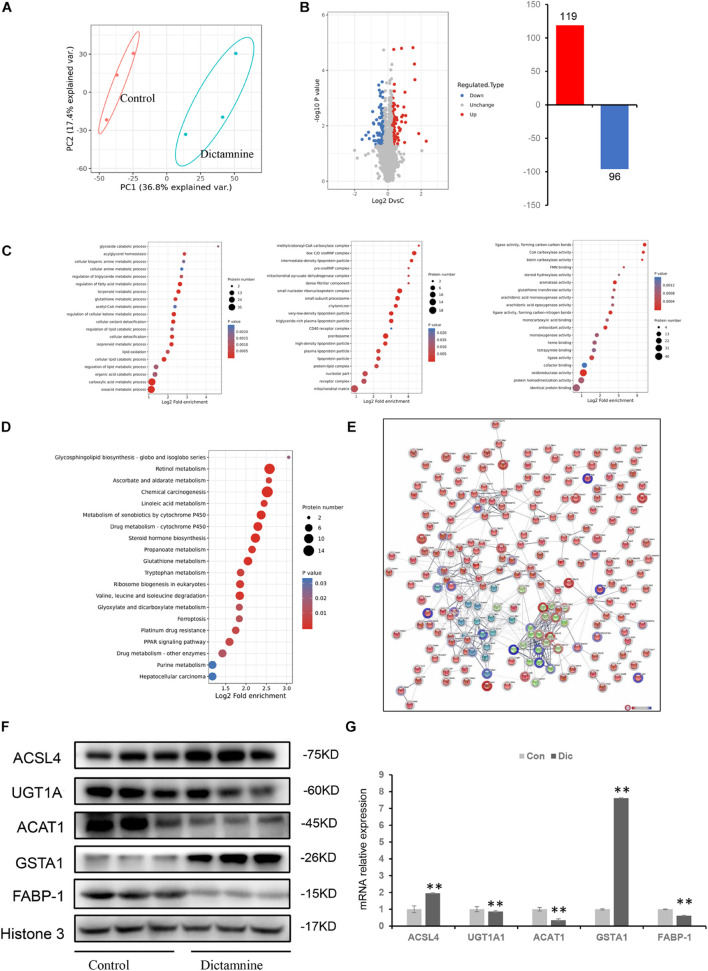
Proteomic analyses of livers of dictamnine-treated mice and untreated control. **(A)** PCA score plot for the differentially expressed proteins in control and dictamnine group. **(B)** The volcano plot (log2 Fold Change vs. –log10 *P*-value) of the up-regulated and down-regulated proteins dictamnine-treated mice and the untreated control. **(C)** Enrichment analysis of GO terms for the differentially expressed proteins. **(D)** KEGG pathway analysis for the differentially expressed proteins in livers of dictamnine-treated mice and untreated control. **(E)** Network analysis for the enriched pathways and protein-protein interactions. **(F)** Western blot analysis of protein expression levels of ACSL4, UGT1A1, ACAT1, and GSTA1 between the two groups. Densitometric analysis shows protein levels relative to Histone 3. **(G)** qRT-PCR analysis of proteins expression levels of ACSL4, UGT1A1, ACAT1, FABP-1, and GSTA1. Values presented as means ± SD (*n* = 3), ^∗∗^*P* < 0.01, *vs.* Con.

To obtain a comprehensive understanding of dictamnine-related HILI, liver tissues from female mice treated with a high-dose dictamnine and the controls were prepared for proteomics analysis based on stringent criteria. After a comparative analysis using the student’s *t*-test, proteins whose abundances ranged from >1.2 to <0.83-fold and *P*-values < 0.05 were selected. A total of 215 proteins were differentially expressed in dictamnine treated groups vs. controls, among which 119 were up-regulated and 96 down-regulated (Figures 4A,B). Gene ontology enrichment analysis revealed that up to 20 differentially expressed proteins were associated with biological process, cellular component, and molecular function GO terms (Figure 4C). The KEGG analysis was further carried out to identify the biological pathways associated with dictamnine-induced hepatotoxicity, to clarify the hepatotoxicity mechanism of dictamnine. The differentially expressed proteins were involved in several processes including, glycosphingolipid biosynthesis, retinol metabolism, linoleic acid metabolism, steroid hormone biosynthesis and glutathione metabolism of biological pathways regulating lipid metabolism and oxidative stress responses (Figure 4D). To predict the putative function and relationship among the differentially expressed proteins, they were imported to the STRING database for protein-protein interaction (PPI) network constructions. Network analysis further revealed a complex functional relationship among the 215 differentially expressed proteins (Figure 4E). However, some proteins such as ACSL4, GSTA1, ACAT1, UGT1A1 and FABP-1 did display any linkage at a confidence level of string score.

### Validation of Differentially Expressed Proteins Linked to Dictamnine-Related Hepatotoxicity by qRT-PCR and Western Blot

Five differentially expressed proteins of interest were selected for confirmation using western blot and qRT-PCR techniques to validate the proteomic analysis results. Of the five proteins, ACAT1, UGT1A1, and FABP-1 were significantly down-regulated, while GSTA1and ACSL4 were significantly up-regulated (Figure 4F). These expression levels were consistent with the protein profiles in TMT analysis. Verification of the mRNA levels of the differentially expressed proteins using qRT-PCR revealed that the mRNA levels of ACAT1, UGT1A1, and FABP-1 were down-regulated, while that of GSTA1and ACSL4 were up-regulated (Figure 4G). These results suggest that the dictamnine-related HILI mechanism may involve oxidative stress, apoptosis, and lipid metabolism disorders.

### Dictamnine-Related Hepatotoxicity Was Associated With Oxidative Stress, Cell Apoptosis and Lipid Metabolism

Dictamnine induces hepatotoxicity in mice, which CYP3A4 modulates through the metabolization of dictamnine (Shi et al., 2019). A significant increase in serum ALT and AST activity was recorded in dictamnine-treated mice compared to the untreated controls (Supplementary Figure 3). However, ALT and AST activity elevation in dictamnine-treated mice was significantly weakened when the mice were pretreated with KTZ, a CYP3A4 inhibitor (Supplementary Figure 3). Thus, while a high dose of dictamnine induced hepatotoxicity, CYP3A4 enzyme activity attenuated the dictamnine-related HILI.

**FIGURE 5:**
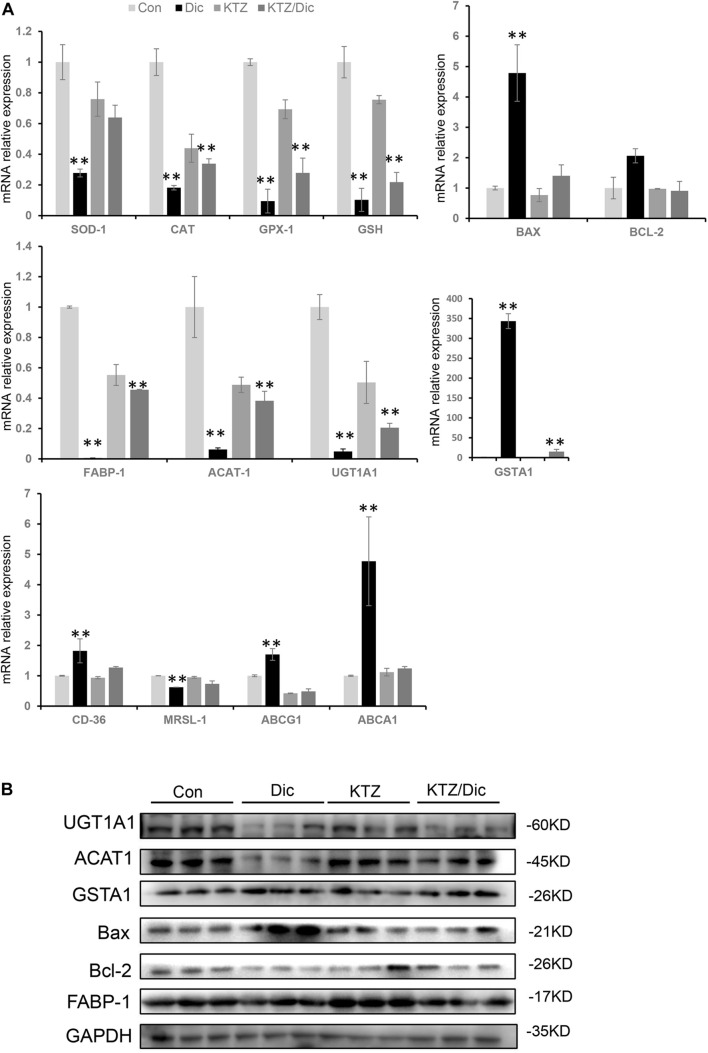
Validation of differentially expressed genes at mRNA and protein levels. **(A)** qRT-PCR analysis of the relative expression of mRNA and protein-related genes. Expression level presented as means ± SD (*n* = 3), ^∗∗^*P* < 0.01, vs. Con. **(B)** Western blot analysis of UGT1A1, ACAT1, and GSTA1, Bax, Bcl-2 and FABP-1 protein levels. Densitometric analysis shows protein levels relative to GAPDH.

Drug-induced liver injury-related oxidative stress was verified by qRT-PCR and western blot analysis. In dictamnine-treated mice, the relative mRNA expression level of GSH, SOD, CAT, and GPx-1 was significantly decreased while that of GSTA1 was significantly increased compared to the control. However, in dictamnine-treated mice pretreatment with KTZ, the decline in GSH, SOD, CAT, and GPx-1 expression levels was relaxed to some extent (Figure 5).

In addition, the Bax to Bax/Bcl-2 mRNA and protein expression ratio was significantly increased in dictamnine-treated mice compared to the control. However, KTZ pretreatment significantly decreased the dictamnine-induced Bax up-regulation to a level equal to the expression in control.

In lipid metabolism, dictamnine inhibited the expression of ACAT1, a key gene for intracellular cholesterol esterification. In addition, the mRNA expression levels of scavenger receptor A (SR-A) and scavenger receptor B (SR-B) were significantly down-regulated while ABCA1 and ABCG1 were significantly increased in the dictamnine-treated group at *P* < 0.05. In dictamnine-treated mice pretreated with KTZ, ACAT1, SR-A and SR-B were significantly up-regulated, while the ABCA1 and ABCG1 expressions were significantly declined (Figure 5).

## Discussion

Herbal remedies have remained popular, with their status sustained more often by traditional experiences than the evidence supporting their efficacy. In recent years, cases of adverse reactions have gradually increased with the increased usage of TCM to treat various diseases (Liu et al., 2020). For example, *Polygonum multiflorum* (PM), which was traditionally considered non-toxic, has been reported to cause serious adverse effects and deaths, raising concerns on the safety of Chinese traditional medicines (Wang et al., 2015).

Dictamni Cortex, the dried root cortex of *Dictamnus dasycarpus* Turcz, is a widely used herbal medicine in many East Asian and European countries. According to Pharmacopoeia of the People’s Republic of China and the principles of TCM, DC is used to clear heat and dispel dampness, dispel pathogenic wind, remove toxins and treat several diseases, including eczema, rubella, tinea, scabies, skin inflammation, acute rheumatism arthritis and jaundice.

Single Chinese herbal medicine is rarely used to treat diseases, while TCM compounds preparation are the main form of TCM used to treat diseases. Examples include the *Zhi-Xue* capsules, derived from the traditional prescription of famous old Chinese medicine, which is among the TCM prescriptions is composed of ethanolic extracts of DC and Radix Sophorae flavescentis. The rigorous phase II and III RCT clinical trial was completed, which was approved by the CFDA for the treatment of hematochezia and rectal tenesmus caused by haemorrhoid in 2003. Herb-induced liver injury caused by DC is not only attributed to the sole use of this herb but also the DC preparation. Unfortunately, according to the National Center for Adverse Drug Reactions Monitoring and related studies in recent years, the Chinese patented medicines “Zhi-Xue capsules” approved by CFDA and compounds derived from DC risks liver injury (Tai et al., 2010). The severe hepatotoxicity of *Zhi-Xue* capsules led to its safety risk warning by the CFDA and was withdrawn from the market in 2008. Furthermore, Wang reported that the clinical cases of 30 patients with *Zhi-Xue* capsules-induced liver injury, 19 and 10 cases recorded a RUCAM score above 8 and 6–8, respectively, indicating causality gradings of highly probable and probable, respectively (Wang et al., 2009). Still, the prevention, diagnosis, and treatment of DC-induced hepatotoxicity remain challenging due to the lack of specific DC-induced hepatotoxicity biomarkers and limited understanding of DC hepatotoxicity mechanisms.

Drug-induced liver injury is classified as either intrinsic or idiosyncratic (Hoofnagle and Bjornsson, 2019). Intrinsic hepatotoxicity refers to the direct toxic stress leading to hepatic cells death associated with a predictable liver injury that can be reproduced in animal models in a dose-dependent manner. Water and ethanol extracts of DC have potential hepatotoxicity in sub-chronic toxicity studies, with the alcohol extraction process increasing the toxicity level (Fan et al., 2018). In our *in vitro* and *in vivo* study, dictamnine was a possible toxic component by high content analysis. For the first time, it was confirmed that mice treated with dictamnine (15mg/kg) for 4 weeks cause hepatotoxicity in female mice, elevating the serum AST activity, liver/brain weight ratio and induce liver histological changes. The finding is consistent with *in vitro* and *in vivo* studies on mice and rats which revealed that the oral administration of DC aqueous extract cause sub-chronic toxicity, which alters the haematological and liver functions due to the metabolic activation of furan, which makes dictamnine hepatotoxic (Wang et al., 2014). Thus, the HILI in our study was considered intrinsic based on previous related *in vivo* and *in vitro st*udies.

Furthermore, the omics (transcriptomic, metabolomics, and proteomics) were used to analyze the serum and tissues in dictamnine-induced HILI. Genome-wide transcriptome analysis established that dictamnine influences the gene expression in *in vitro* and *in vivo* studies, significantly disrupting the lipid metabolism and accelerating the oxidative stress, with the effects being more pronounced in female mice than their male counterparts. These findings are consistent with the phenotypic assays. The calculated differentially expressed genes in dictamnine-treated female and male mice correlated with those expressed in the cellular transcriptome. Two groups (Con vs. Dic) were distinctly identified on the PCA scores scatter plots by metabolomics analysis (Figure 4A). These results suggest a potential disorder on the host biological network following the dictamnine-induced hepatotoxicity.

The KEGG pathway analysis revealed that the differentially expressed metabolites were mainly involved in the primary bile acid biosynthesis, sphingolipid metabolism, glycerophospholipid metabolism, etc. Among the differentially expressed metabolites, chenodeoxycholate, taurocholate, and cholate presented excellent discrimination between the study groups (AUC ≥ 0.9, *P* < 0.01), hence have great potential for use as biomarkers of dictamnine-induced hepatotoxicity. These biomarkers increase the understanding of DC-induced hepatotoxicity mechanisms and can promote clinically safe usage of DC.

However, very few studies have evaluated the use of biomarkers to assess HILI caused by DC toxicity in humans; thus, the direct and reliable research evidence on this issue for reference is lacking. Fortunately, the use of biomarkers to assess the susceptibility to *Polygonum multiflorum*-HILI has been documented in previous research (Tu et al., 2019, 2021). Therefore, it is of significant clinical significance to validate the identified biomarkers in our animal experiments to establish their efficacy as indicators of DC related HILI.

Most of the differentially expressed genes in the transcriptome were not expressed in the proteome and vice versa. This can be attributed to the set experimental timeliness and other reasons. Therefore, genes and proteins with changes in both the transcriptome and proteome were selected for subsequent verification. Additionally, three down-regulated proteins (ACAT1, UGT1A1, and FABP-1) and two up-regulated proteins (GSTA1 and ACSL4) predicted by the proteomic analysis were verified by western blot. Differentially expressed proteins were mainly involved in metabolic pathways, especially glutathione metabolism, linoleic acid metabolism and glycosphingolipid biosynthesis, which suggests that the mechanisms of dictamnine-induced hepatotoxicity are highly associated with lipid metabolism and oxidative stress. The hepatotoxicity mechanisms of dictamnine involve increasing the initial oxidative stress through the up-regulation of GSTA1, exhaustion of GSH, and decreasing the down-regulation of antioxidative enzymes (SOD, CAT and GPx-1). When the hepatic GSH reserves are exhausted, the reactive metabolites bind to functional proteins or other nucleophiles, causing cell apoptosis (Yang et al., 2018). In addition, our results revealed that dictamnine up-regulated Bax and down-regulated Bcl-2 proteins in the liver, which is consistent with the findings in previous studies (Fan et al., 2018).

Dictamnine is a major furoquinoline alkaloid component in DC which is metabolized into an intermediate epoxide (2,3-epoxide) by CYP3A4 to prevent DC-related HILI (Li et al., 2018; Shi et al., 2019). In the present study, pretreatment with KTZ, an inhibitor of the CYP3A4 enzyme, alleviated the hepatotoxicity level induced by dictamnine. KTZ pretreatment reduces liver damage by initially moderating oxidative stress followed by Bax/Bcl-2 expression inhibition (Figure 5). Thus, oxidative stress-induced apoptosis is dominant in the early stages of dictamnine-induced hepatotoxicity. KTZ prevents hepatotoxicity by decreasing oxidative stress through inhibiting Bax/Bcl2-independent pathway.

Abnormal lipid metabolism in macrophages leads to various diseases, including various liver diseases, atherosclerosis, etc. (Tall and Yvan-Charvet, 2015). This happens due to the altered regulation of lipid metabolism caused by the disturbance of intracellular cholesterol influx and efflux and the intracellular cholesterol metabolism (Shao et al., 2020). Therefore, regulatory proteins such as ACAT1, SR-A and ABCA1, which mediate processes in the cholesterol metabolism pathway, are critical in regulating macrophage cholesterol accumulation. In this study, dictamnine remarkably inhibited the expression of ACAT1, a key enzyme in maintaining lipid metabolism balance by catalyzing the formation of cholesterol esters from free cholesterol and long-chain fatty acids (Moore and Tabas, 2011). Consequently, dictamnine inhibited the process of cholesterol ester formation from free cholesterol by suppressing the expression of ACAT1. In macrophages, this increased free cholesterol is transported out of the cells to the liver for further metabolic transformation. Cholesterol is the major precursor in bile acid synthesis via two major bile acid biosynthetic pathways. In our study, the accumulation of bile acids (chenodeoxycholate, taurocholate, and cholate) was significantly increased in dictamnine treated group. Accumulation of bile acids in the liver causes hepatic inflammation and injury (Li and Apte, 2015). As a result, patients with cholestasis develop fibrosis, cirrhosis, increased risk of hepatocellular carcinomas and eventually liver failure over time (McLaren et al., 2011).

Besides, dictamnine-induced FABP-1 inhibition potentially affected the metabolism of the fatty acids. FABP1 plays an important role in lipid uptake and transport in the liver. Therefore, the down-regulation of FABP1 could enhance lipotoxicity of free fatty acids, contributing to inflammation, steatohepatitis and non-alcoholic fatty liver disease (NAFLD) progression (Neuschwander-Tetri, 2010; Guzmán et al., 2013). In addition, the ACSL4 mRNA and protein levels were increased in the dictamnine-induced hepatotoxicity group. Acyl-CoA synthetase long-chain family member 4 (ACSL4), a key enzyme that regulates lipid composition, and also contributes to ferroptosis (Doll et al., 2017; Li et al., 2019). Thus, dictamnine inhibits the ACAT1 and FABP-1expression and activates ACSL4 expression, which collectively contributes to lipid metabolism disorder and the progression of hepatotoxicity.

## Conclusion

The findings in our study demonstrated that dictamnine cause dose-dependent hepatotoxicity in female mice after a chronic exposure leading to liver injury characterized by hepatocyte steatosis and macrovesicles. Dictamnine-induced hepatotoxicity mechanisms involved increased oxidative stress and early hepatic apoptosis. This occurred via the up-regulation of GSTA1, down-regulation of antioxidative enzymes such as SOD, CAT, and GP_*X*_-1, and increased Bax/Bcl-2 ratio. Dictamnine also increased intracellular lipid metabolism disorder via the down-regulation of ACAT1 and FABP-1 and up-regulation of ACSL4. This study provides references for the development and evaluation of new Chinese medicines containing DC components. The findings also provide a basis for the formulation and improvement of quality and safety control standards for DC-containing medication and the rational use of DC in clinical settings, especially when administering DC for a long time. It is recommended to monitor the liver function regularly, consider the benefits and risks of treatment, and advise the patients accordingly.

## Data Availability Statement

The original contributions presented in the study are included in the article/Supplementary Material, further inquiries can be directed to the corresponding authors.

## Ethics Statement

The animal study was reviewed and approved by the Experimental Animal Ethics Subcommittee of Beijing University of Chinese Medicine.

## Author Contributions

CT and ZX performed the experiments, analyzed the data, and wrote the manuscript. LT and ZG performed data analysis and discussed the corresponding mechanism. JZ and TinW conceived and led the project. TinW supported and supervised the research. All authors made contribution to technical discussions and manuscript writing.

## Conflict of Interest

The authors declare that the research was conducted in the absence of any commercial or financial relationships that could be construed as a potential conflict of interest.

## Publisher’s Note

All claims expressed in this article are solely those of the authors and do not necessarily represent those of their affiliated organizations, or those of the publisher, the editors and the reviewers. Any product that may be evaluated in this article, or claim that may be made by its manufacturer, is not guaranteed or endorsed by the publisher.

## Supplementary Material

Supplementary Figure 1 RNA-seq analysis *in vitro* and *vivo*. **(A)** GO analysis of RNA-seq data of HepaRG cells. **(B)** The volcano map of the differentially expressed genes in dictamnine-treated and untreated control mice. **(C)** Enriched GO terms of RNA-seq of male and female ICR mice. **(D)** Correlation and clustering of RNA-seq data of male and female ICR mice. **(E)** Enriched KEGG terms of RNA-seq of dictamnine-treated and untreated female ICR mice. **(F)** The top enriched KEGG terms of RNA-seq from dictamnine treated and untreated female ICR mice.

Supplementary Figure 2 ROC curves of the differentially expressed metabolites. **(A)** Receiver-operating-characteristic (ROC) curve showing the area under the curve (AUC) for the 28 differentially expressed metabolites between the control (Con) and dictamnine (Dic) group. The color indicates the value of AUC. **(B)** ROC curves for the 13 differentially expressed metabolites between dictamnine-treated mice and untreated control. **(C)** Quantification of the high value biomarkers (^∗^*P* < 0.05, ^∗∗^*P* < 0.01, *vs.* Con.).

Supplementary Figure 3 The serum ALT and AST activities of dictamnine-induced liver injury in mice.

## Abbreviations

HILI: Herb induced liver injury
DC: Dictamni Cortex
TCM: Traditional Chinese Medicine
RUCAM: Roussel Uclaf Causality Assessment Method
ALT: Alanine aminotransferase
AST: Aspartate aminotransferase
DBIL: Direct bilirubin
TBIL: Total bilirubin
CFDA: Chinese Food and Drug Administration
PCA: Principal component analysis
ROC: Receiver operating characteristic
SOD: superoxide dismutase
CAT: Catalase
GPx-1: Glutathione peroxidase 1
ROS: reactive oxygen species
HE: Hematoxylin-eosin
ACAT1: Acetyl-coa acetyltransferase 1
ACSL4: Acyl-CoA synthetase long-chain family member 4
UGT1A1: Uridine diphosphate-glucuronosyl- transferase1A1
GSTA1: Glutathione S transferase A1
FABP1: Fatty acid binding protein 1
DEHP: Di-(2-ethylhexyl) phthalate
HCC: hepatocellular carcinomas.
