# Supplementary material for: Multi-Omics Integration to Reveal the Mechanism of Hepatotoxicity Induced by Dictamnine
Source: Front Cell Dev Biol. 2021 Sep 14;9:700120. doi: 10.3389/fcell.2021.700120 (PMC8476863; doi:10.3389/fcell.2021.700120)
Supplement: Supplementary Figure 1 — RNA-seq analysis in vitro and vivo. (A) GO analysis of RNA-seq data of HepaRG cells. (B) The volcano map of the differentially expressed genes in dictamnine-treated and untreated control mice. (C) Enriched GO terms of RNA-seq of male and female ICR mice. (D) Correlation and clustering of RNA-seq data of male and female ICR mice. (E) Enriched KEGG terms of RNA-seq of dictamnine-treated and untreated female ICR mice. (F) The top enriched KEGG terms of RNA-seq from dictamnine treated and untreated female ICR mice. [file Data_Sheet_2.docx]

**1. Supplemental Figures**


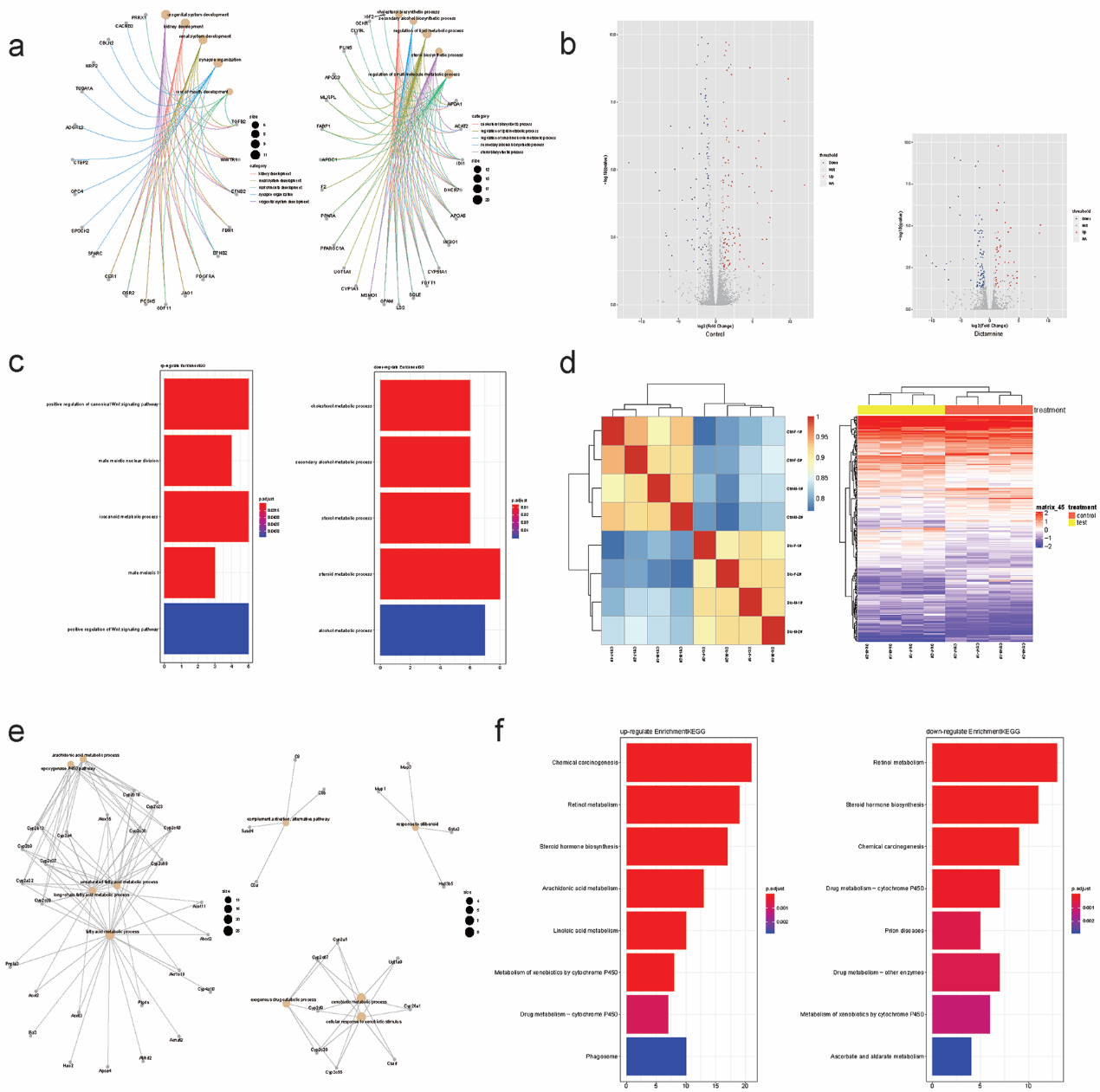


**Supplemental Figure S1. RNA-seq analysis *in vitro and vivo*.** **a**. GO analysis of RNA-seq data of HepaRG cells. **b**. The volcano map of the differentially expressed genes in dictamnine-treated and untreated control mice. **c.** Enriched GO terms of RNA-seq of male and female ICR mice. **d**. Correlation and clustering of RNA-seq data of male and female ICR mice. **e.** Enriched KEGG terms of RNA-seq of dictamnine-treated and untreated female ICR mice. **f**. The top enriched KEGG terms of RNA-seq from dictamnine treated and untreated female ICR mice.


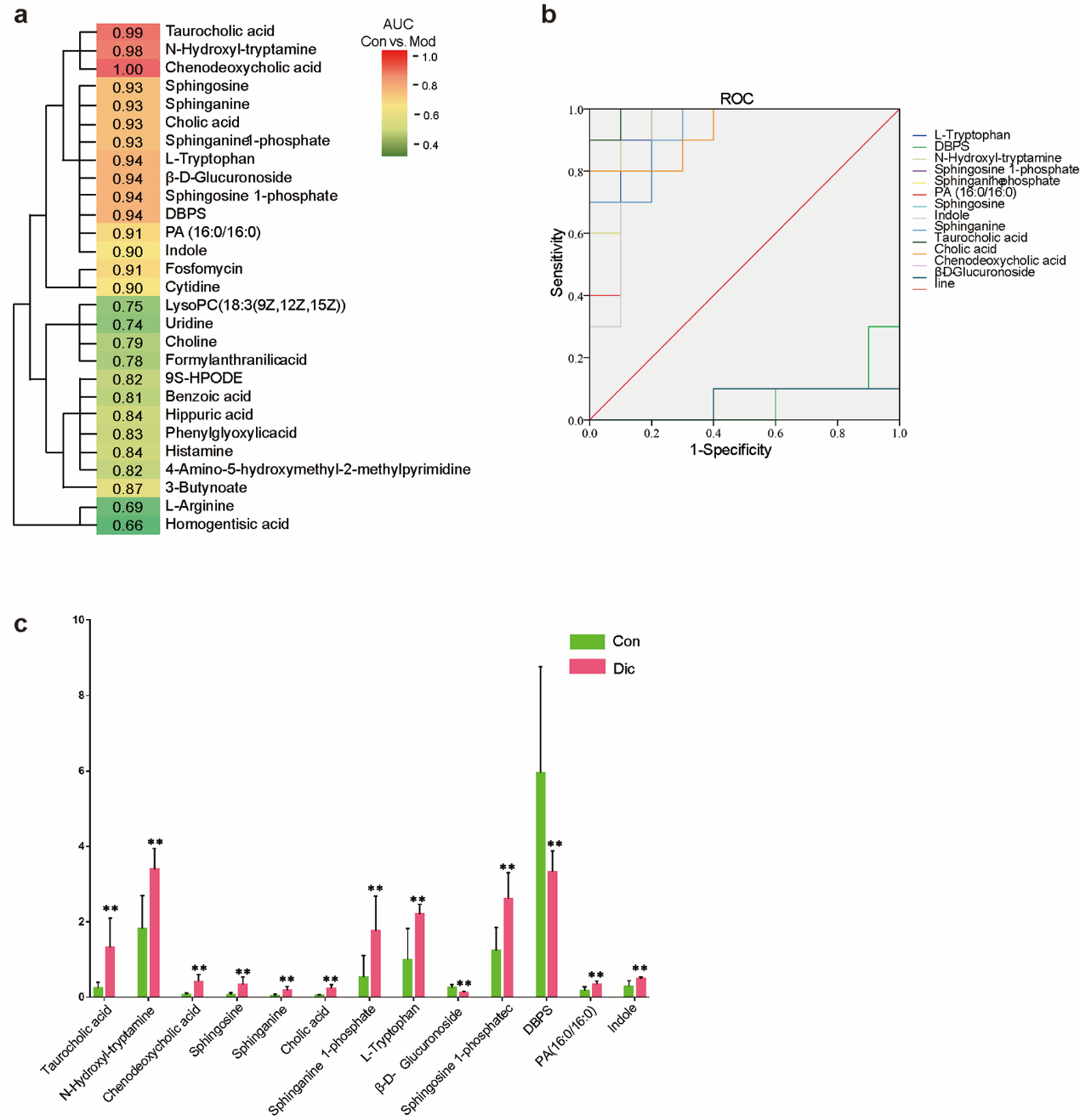


**Supplemental Figure S2. ROC curves of differentially expressed metabolites.** **a**. Cluster analysis on the area under curve (AUC) and P values of the receiver-operating-characteristic curve (ROC) of each 28 metabolites in discriminating the control (Con) vs dictamnine (Dic) group. The color indicates the value of AUC. **b**. ROC curves of 13 metabolites in discriminating the dictamnine group from the control group. **c**. Content changes of high value identified biomarkers, such as chenodeoxycholate, taurocholate and cholate, etc. (**P* < 0.05, ***P* < 0.01 vs. control group).


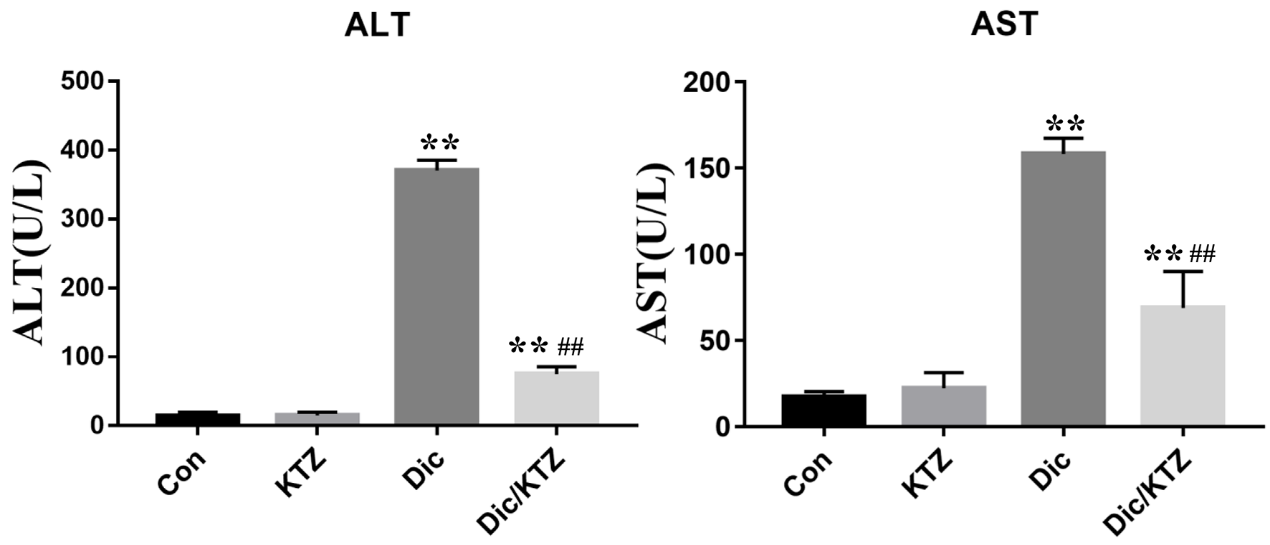


**Supplemental Figure S3. The serum ALT and AST activities of dictamnine-induced liver injury in mice.** (**P* < 0.05, ***P* < 0.01 *vs.* control group；^##^ *P* < 0.01 *vs.* Dic group)**.**

**2. Supplemental Tables**

**Supplemental Table S1: Antibodies source**

| Protein | Source | Dilution | Cat# |
| --- | --- | --- | --- |
| GAPDH | Proteintech | 1:3000 | 60004-1-lg |
| Acetyl-CoA acetyltransferase, mitochondrial  （ACAT1） | Abcam | 1:1000 | Ab168342 |
| Glutathione S-transferase A1  （GSTA1） | Proteintech | 1:20000 | 66624-1-lg |
| UDP-glucuronosyltransferase 1-1  (UGT1A1) | Abcam | 1:1000 | Ab194697 |
| Fatty acid-binding protein 1(FABP-1) | Cell Signaling | 1:1000 | 13368T |
| Bax | Abcam | 1:2000 | ab3191 |
| Bcl-2 | Abcam | 1:2000 | ab218123 |
| Histone | Proteintech | 1:1000 | 17168-1-AP |
| acyl-CoA synthetase long-chain family member 4(ACSL4) | Santa cruz | 1:1000 | sc-365230 |

**Supplemental Table S2: The primer sequence for qRT-PCR**

| Genes |  | Sequences (5’ to 3’) | |
| --- | --- | --- | --- |
| GAPDH | Sense | AGGTCGGTGTGAACGGATTTG |  |
|  | Antisense | GGGGTCGTTGATGGCAACA |  |
| GSTA1 | Sense | AAGCCCGTGCTTCACTACTTC | |
|  | Antisense | GGGCACTTGGTCAAACATCAAA | |
| UGT1A1 | Sense | CACCTGAAGCCTCAATACACAT | |
|  | Antisense | CAGTCCGTCCAAGTTCCACC | |
| ACAT1 | Sense | TGTAAAAGACGGGCTAACTGATG | |
|  | Antisense | TGTTCCTGCCGTGAGATATTCAT | |
| FABP-1 | Sense | ATGAACTTCTCCGGCAAGTACC | |
|  | Antisense | CTGACACCCCCTTGATGTCC | |
| ABCA1 | Sense | AAGGATTTCTTATCGTGCAGCA | |
|  | Antisense | CTCCGCATAAATTGTGGTGTTG | |
| BCL-2 | Sense | GAGAGCGTCAACAGGGAGATG | |
|  | Antisense | CCAGCCTCCGTTATCCTGGA | |
| BAX | Sense | TGAAGACAGGGGCCTTTTTG | |
|  | Antisense | AATTCGCCGGAGACACTCG | |
| SOD1 | Sense | AACCAGTTGTGTTGTCAGGAC | |
|  | Antisense | CCACCATGTTTCTTAGAGTGAGG | |
| CAT | Sense | GGAGGCGGGAACCCAATAG | |
|  | Antisense | GTGTGCCATCTCGTCAGTGAA | |
| GP_X_-1 | Sense | AGTCCACCGTGTATGCCTTCT | |
|  | Antisense | GAGACGCGACATTCTCAATGA | |
| ASCL4 | Sense | CCTGAGGGGCTTGAAATTCAC | |
|  | Antisense | GTTGGTCTACTTGGAGGAACG | |
| CD36 | Sense | AGATGACGTGGCAAAGAACAG | |
|  | Antisense | CCTTGGCTAGATAACGAACTCTG | |
| SR-A | Sense | AAGTATCAGCAGAAGTCCAGTCT | |
|  | Antisense | TCCTTCAGTCTGAGGTCGTTG | |
| ABCG1 | Sense | GCTCCATCGTCTGTACCATCC | |
|  | Antisense | ACGCATTGTCCTTGACTTAGG | |

**Supplemental Table S3: Effects of oral administration of dictamnine on liver-related indexes in mice**

| **Group** | **Female (Dic, mg/kg)** | | | | **Male (Dic, mg/kg)** | | | |
| --- | --- | --- | --- | --- | --- | --- | --- | --- |
|  | Control | 15 | 3 | 0.6 | Control | 15 | 3 | 0.6 |
| Body weight(g) | 29.26±2.13 | 28.02±2.26 | 27.30±0.71 | 28.99±1.67 | 37.27±2.62 | 33.69±1.44 | 34.52±2.10 | 37.36±1.63 |
| Absolute liver weight(g) | 1.14±0.06 | 1.32±0.13 | 1.16±0.06 | 1.23±0.17 | 1.68±0.20 | 1.68±0.15 | 1.65±0.20 | 1.84±0.17 |
| Relate liver  weight (%) | 3.89±0.12 | 4.79±0.20** | 4.25±0.17** | 4.05±0.29 | 4.44±0.30 | 5.00±0.37** | 4.76±0.47 | 4.92±0.41* |
| Liver/brain (%) | 2.51±0.21 | 2.89±0.25** | 2.50±0.19 | 2.58±0.33 | 3.66±0.31 | 3.77±0.31 | 3.60±0.36 | 3.97±0.40 |

Values are expressed as mean ± SD; n = 10; * *P* < 0.05, ** *P* < 0.01, compared with the control group.

**Supplemental Table S4: Identified differential metabolites between dictamnine and control group in female mice**

| No. | M/Z | TR (min) | Metabolites | Metabolite pathways | VIP | FC | *P* value |
| --- | --- | --- | --- | --- | --- | --- | --- |
| **ESI^+^** |  |  |  |  |  |  |  |
| 1 | 174.1120 | 0.765 | L-Arginine | Arginine and proline metabolism | 2.82694 | 0.4680 | 0.0443 |
| 2 | 84.0216 | 1.033 | 3-Butynoate | Butanoate metabolism | 1.72102 | 2.5753 | 0.0064 |
| 3 | 103.1002 | 18.741 | Choline | Glycerophospholipid metabolism | 1.38658 | 0.4200 | 0.0146 |
| 4 | 434.2438 | 16.847 | PA (16:0/16:0) | Glycerophospholipid metabolism | 1.25982 | 2.6660 | 0.0006 |
| 5 | 517.3145 | 16.608 | LysoPC (18:3(9Z,12Z,15Z)) | Glycerophospholipid metabolism | 5.71356 | 0.3481 | 0.0338 |
| 6 | 111.0799 | 20.413 | Histamine | Histidine metabolism | 1.47243 | 0.2662 | 0.0436 |
| 7 | 122.0334 | 0.719 | Benzoate | Phenylalanine metabolism | 3.11885 | 3.3326 | 0.0070 |
| 8 | 150.0282 | 0.721 | Phenylglyoxylic acid | Phenylalanine metabolism | 3.58357 | 4.1719 | 0.0042 |
| 9 | 138.0107 | 20.949 | Fosfomycin | Phosphonate and phosphinate metabolism | 3.23876 | 0.4424 | 0.0111 |
| 10 | 243.0802 | 20.89 | Cytidine | Pyrimidine metabolism | 2.98604 | 0.4396 | 0.0127 |
| 11 | 244.0666 | 20.877 | Uridine | Pyrimidine metabolism | 1.04583 | 0.4421 | 0.0369 |
| 12 | 184.0162 | 20.997 | 3,4-dihydroxy-2-butanone 4-phosphate synthase | Riboflavin metabolism | 5.30152 | 0.4514 | 0.0097 |
| 13 | 139.0747 | 20.677 | 4-Amino-5-hydroxymethyl-2-methylpyrimidine | Thiamine metabolism | 1.00306 | 0.4061 | 0.0315 |
| 14 | 117.0582 | 4.204 | Indole | Tryptophan metabolism | 1.50931 | 2.4012 | 0.0008 |
| 15 | 165.0462 | 1.077 | Formylanthranilic acid | Tryptophan metabolism | 1.02282 | 2.3544 | 0.0192 |
| 16 | 176.0952 | 1.633 | N-Hydroxyl-tryptamine | Tryptophan metabolism | 3.77276 | 2.3161 | 0.0001 |
| 17 | 204.0902 | 3.507 | L-Tryptophan | Tryptophan metabolism | 11.3472 | 3.6724 | 0.0003 |
| 18 | 168.0386 | 20.875 | Homogentisate | Tyrosine metabolism | 1.11792 | 0.4706 | 0.0457 |
| 19 | 299.2826 | 15.639 | Sphingosine | Sphingolipid metabolism | 1.38311 | 8.4645 | 0.0005 |
| 20 | 301.2983 | 16.126 | Sphinganine | Sphingolipid metabolism | 1.09542 | 18.1120 | 0.0004 |
| 21 | 379.2491 | 14.605 | Sphingosine 1-phosphate | Sphingolipid metabolism | 3.68197 | 3.0750 | 0.0002 |
| 22 | 381.2648 | 14.998 | Sphinganine 1-phosphate | Sphingolipid metabolism | 3.21973 | 7.9630 | 0.0021 |
| **ESI^-^** |  |  |  |  |  |  |  |
| 1 | 179.0578 | 5.023 | Hippurate | Phenylalanine metabolism | 2.10089 | 15.1940 | 0.0149 |
| 2 | 312.2312 | 16.101 | 9(S)-HPODE | Linoleic acid metabolism | 1.29797 | 2.9072 | 0.0140 |
| 3 | 392.2941 | 14.88 | Chenodeoxycholate | Primary bile acid biosynthesis | 1.52521 | 5.6549 | 0.0000 |
| 4 | 408.2889 | 11.212 | Cholate | Primary bile acid biosynthesis | 1.08573 | 4.7533 | 0.0000 |
| 5 | 515.2932 | 10.189 | Taurocholate | Primary bile acid biosynthesis | 2.5296 | 5.9022 | 0.0004 |
| 6 | 606.3300 | 15.902 | β-D-Glucuronoside | Pentose and glucuronate interconversions | 1.35946 | 0.4178 | 0.0004 |
