# Supplementary material for: Multi-Omics Integration to Reveal the Mechanism of Hepatotoxicity Induced by Dictamnine
Source: Front Cell Dev Biol. 2021 Sep 14;9:700120. doi: 10.3389/fcell.2021.700120 (PMC8476863; doi:10.3389/fcell.2021.700120)
Supplement: Supplementary file 2 [file Data_Sheet_1.DOCX]

**Multi-omics integration to reveal the mechanism of hepatotoxicity induced by dietary supplement dictamnine**

Can Tu^1,†^, Ziying Xu^1,^**^†^**, Lichun Tian^1^, Zihui Yu^2^, Tieshang Wang^1^, Zhaojuan Guo^1^,

Jingxuan Zhang^1,^*, Ting Wang^1,^*

^1^ Beijing Research Institute of Chinese Medicine, Beijing University of Chinese Medicine, Beijing 100029, China

^2^ Key Laboratory of Genomics and Precision Medicine, Beijing Institute of Genomics, Chinese Academy of Sciences, Beijing 100101, China

**^†^ These authors have contributed equally to this work**

# Materials and Methods

## RNA-seq

Cells and Liver tissues were disrupted in liquid nitrogen, and total RNA was prepared using Trizol Reagent (Invitrogen, USA) according to the manufacturers’ instructions. The Ribo-off rRNA Depletion Kit and VAHTS Universal V6 RNA-seq Library Preparation Kit was used to construct stranded RNA-seq libraries according to the manufacturer’s instructions.

## RNA-seq and gene set enrichment analysis

Two biological replicates were performed in each treatment situation. Trimomatic (version 0.36) discarded the reading with length less than 35 nt or containing ambiguous nucleotides. The remaining readings were consistent with human reference genome (hg19) and mouse reference genome (mm10) using HISAT2. For each sample, only the unique map reads with map quality score ≥ 20 were reserved for subsequent analysis. The HT Seq Python package (version 0.9.1) was used to count the number of reads of a unique map for each gene. R-package DEseq2 was used to determine the differentially expressed genes between the treatment group and the control group, fold-change cutoff= 1.0, p value cut off= 5× 10^−2^. R-package of maSigPro was used to analyze the time series and determine the differentially expressed genes with the duration of the medication, Q = 0.05, MT. adjust = "BH", alfa = 0.05.R-package Cluster Profiler was used to analyze Gene ontology (GO) of genes for functional enrichment analysis, only p<0.05 GO terms were retained.

## Chromatography and Mass Spectrometry Conditions

UPLC analysis was performed on a Thermo Scientific Dionex UltiMate 3000 UHPLC system (Santa Clara, CA, USA) equipped with an autosampler, a vacuum degasser unit, a quaternary pump, a diode array detector (DAD) and a column compartment. Samples were separated on an ACQUITY UPLC BEH-C18 column (2.1mm×100 mm, 1.7 μm) at 35 °C. The mobile phase consisted of acetonitrile (A) and 0.1% formic acid water (B), and the elution gradient was set as follows: 0~1 min, 5% A; 1~9 min,5%~40% A; 9~19 min, 40%~90% A; 19~24 min,90%~5% A. The flow rate was set at 0.30 mL/min, and the injection volume was 4 μL.

ESI-MS analyses were performed on a Thermo Scientific Q Exactive Plus LC-MS mass spectrometer (Santa Clara, CA, USA) equipped with an electrospray ionization interface and a hesi-ii source. Samples were analyzed in negative ion mode, and full-scan mass spectra were acquired in a mass range of m/z 80 to 1500 Da. The resolving power was 30,000 for the full scans and 15,000 for the MS scans. The spray and capillary voltages were set to 4.0 KV and 25.0 V, respectively; the tube lens was set to 110 V; and the source temperature was set to 300 °C. Nitrogen (purity > 99.99%) was used as both the sheath gas (30 units) and auxiliary gas (10 units).

## Identification of the Metabolites and Metabolic Pathway Analysis

Endogenous metabolites that contributed to the classification were found by variable importance in the projection (VIP) values, which showed the importance of each variable to the classification. Only VIP values *>* 1 were selected and used for further data analysis. Compounds with significant changes in the groups (*P*-value *<* 0.05 and fold change *>* 1.5) were selected as candidate biomarkers. All biomarkers were tentatively identified with the accurate mass-to-charge ratio in Mass Hunter PCDL Manager database and KEGG database. The pathways analysis of potential biomarkers was performed with MetaboAnalyst 3.0 based on the pathway library of rattus. The ROC analysis was performed with MetaboAnalyst 3.0 based on the biomarker analysis. Independent sample *t*-test was used to reveal the statistical differences of data between two groups. Statistical differences were considered significant when the *P*-value was lower than 0.05.

## Sample Preparation for Proteome MS Analysis

**Protein extraction, digestion, peptide purification, TMT labeling**

The tissue samples were grinded by liquid nitrogen into cell powder and then transferred to the centrifuge tube. After that, four volumes of lysis buffer (8 M urea, 1% Protease Inhibitor Cocktail) was added to the cell powder, followed by sonication three times on ice using a high intensity ultrasonic processor. The remaining debris was removed by centrifugation at 12,000 g at 4 °C for 10 min. Finally, the supernatant was collected and the protein concentration was determined with BCA kit according to the manufacturer’s instructions.

**Trypsin Digestion**

The protein solution was reduced with 5 mM dithiothreitol for 30 min at 56 °C and alkylated with 11 mM iodoacetamide for 15 min at room temperature in darkness. The protein sample was then diluted by adding 100 mM TEAB to urea concentration less than 2M. Finally, trypsin was added at 1:50 trypsin-to-protein mass ratio for the first digestion overnight and 1:100 trypsin-to-protein mass ratio for a second 4 h-digestion.

**TMT Labeling**

After trypsin digestion, peptide was desalted by Strata X C18 SPE column (Phenomenex) and vacuum-dried. Peptide was reconstituted in 0.5 M TEAB and processed according to the manufacturer’s protocol for TMT kit. Three pairs of control and dictamnine liver samples were labeled with the six channels (control labeled with 126, 127, 128; dictamnine liver tissue labeled with 129, 130 and 131) Briefly, one unit of TMT reagent were thawed and reconstituted in acetonitrile. The peptide mixtures were then incubated for 2 h at room temperature and pooled, desalted and dried by vacuum centrifugation. The liver tissue samples were eventually labeled in 3 sets of TMT experiments for the LC-MS/MS analysis.

**LC-MS/MS** **analysis**

The tryptic peptides were dissolved in solvent A, directly loaded onto a home-made reversed-phase analytical column (15cm length, 75 μm i.d.). The mobile phase, consisting of a mixture of 0.1% formic acid and 2% acetonitrile（A）and 0.1% formic acid and 90% acetonitrile (B). The binary gradient elution protocol was as follows: 0~30min, 8-20% B; 30~52 min, 20-32% B; 52~56 min, 32-80% B; 56~60min, 80% B. all at a constant flow rate of 400 nL/min on an EASY-nLC 1000 UPLC system. The peptides were subjected to NSI source followed by tandem mass spectrometry (MS/MS) in Orbitrap Fusion Lumos coupled online to the UPLC. The electrospray voltage applied was 2.0 kV. The m/z scan range was 350 to 1550 for full scan, and intact peptides were detected in the Orbitrap at a resolution of 60,000. Peptides were then selected for MS/MS using NCE setting as 28 and the fragments were detected in the Orbitrap at a resolution of 15,000. A data-dependent procedure that alternated between one MS scan followed by 20 MS/MS scans with 15.0s dynamic exclusion. Automatic gain control (AGC) was set at 5E4. Fixed first mass was set as 100 m/z.

**Database Search and Bioinformatics Analysis**

The resulting MS/MS data were processed using Maxquant search engine (v.1.5.2.8). The mass tolerance for precursor ions was set as 20 ppm in First search and 5 ppm in Main search, and the mass tolerance for fragment ions was set as 0.02 Da. Proteins were classified by GO annotation into three categories: biological process, cellular compartment, and molecular function. For each category, a two-tailed Fisher’s exact test was employed to test the enrichment of the differentially expressed protein against all identified proteins. The GO with a corrected p-value < 0.05 is considered significant. Encyclopedia of Genes and Genomes (KEGG) database was used to identify enriched pathways by a two-tailed Fisher’s exact test to test the enrichment of the differentially expressed protein against all identified proteins. The pathway with a corrected p-value < 0.05 was considered significant. All differentially expressed protein database accession or sequence were searched against the STRING database version 10.1 for protein-protein interactions. Only interactions between the proteins belonging to the searched data set were selected, thereby excluding external candidates. STRING defines a metric called “confidence score” to define interaction confidence; we fetched all interactions that had a confidence score ≥ 0.7 (high confidence). Interaction network form STRING was visualized in R package “networkD3”.
